# Wearable Stretch Sensors for Motion Measurement of the Wrist Joint Based on Dielectric Elastomers

**DOI:** 10.3390/s17122708

**Published:** 2017-11-23

**Authors:** Bo Huang, Mingyu Li, Tao Mei, David McCoul, Shihao Qin, Zhanfeng Zhao, Jianwen Zhao

**Affiliations:** 1 Department of Mechanical Engineering, Harbin Institute of Technology, Weihai 264209, China; huangbo74@163.com (B.H.); limingyulucy@163.com (M.L.); meitao0101@163.com (T.M.); HITqinshihao@163.com (S.Q.); 2 Department of Materials Science and Engineering, UCLA, Los Angeles, CA 90095, USA; davidmccoul@gmail.com; 3 Department of Electrical Engineering, Harbin Institute of Technology, Weihai 264209, China; zhaozhanfeng@hitwh.edu.cn

**Keywords:** dielectric elastomer sensors, wearable sensors, motion capture, soft sensors

## Abstract

Motion capture of the human body potentially holds great significance for exoskeleton robots, human-computer interaction, sports analysis, rehabilitation research, and many other areas. Dielectric elastomer sensors (DESs) are excellent candidates for wearable human motion capture systems because of their intrinsic characteristics of softness, light weight, and compliance. In this paper, DESs were applied to measure all component motions of the wrist joints. Five sensors were mounted to different positions on the wrist, and each one is for one component motion. To find the best position to mount the sensors, the distribution of the muscles is analyzed. Even so, the component motions and the deformation of the sensors are coupled; therefore, a decoupling method was developed. By the decoupling algorithm, all component motions can be measured with a precision of 5°, which meets the requirements of general motion capture systems.

## 1. Introduction

The wrist is one of the most complex joints of the human body, and plays a very important role in the action of the hand. Motion capture systems for tracking wrist motion are widely applied in many fields. In terms of human-computer interaction, a sensing wristband was used to control the roll, pitch, and yaw of a small quad-copter in [1]; several systems for wrist rehabilitation have been developed in research centers and universities that are applied to rehabilitation in health centers [2,3,4]. In addition, the capture and analysis of joint motions can improve the competitiveness of some sports; for example, badminton requires proper wrist angles to obtain better effectiveness, and accurate feedback of wrist motion can guide training for the sport [5].

There are two main types of traditional sensor systems that have been used to measure wrist motion. One is based on camera and image processing [6], and the other is based on rigid sensors [7]. In the first motion capture system, high-speed wrist movement is captured by a camera and analyzed through an algorithm. However, in this system, cameras need to be placed around the test area, wrist movements must be confined to within this limited space [8], and the complicated image processing decreases the measurement speed. In the second system, accelerometers and gyroscopes can be placed on the wrist to measure its motion via changes in acceleration or inertia. Compared to camera systems, this type of sensor is free from sound and light interference, but suffers from integration drift and the continuous need to re-adjust their absolute position [9,10]. Due to the limitations of the above two systems, research has been focused on wearable systems based on flexible sensors. In one example, a wearable posture recognition system is composed of 21 textile strain sensors, and 27 upper body postures could be recognized with an accuracy of 97% [11]. In addition, the 5DT Data Glove based on five fiber optic sensors is designed to satisfy the requirements of high-precision finger motion capture [12].

Some materials have been investigated for the fabrication of wearable flexible sensors, such as conductive rubber [13,14], conductive fibers/fabrics [15,16,17,18,19], polyvinylidene fluoride (PVDF) [20,21], and nanocomposites [22,23]. Conductive rubber is easy to manufacture and can conduct multi-point measurements, but is mostly used for pressure testing and can exhibit relaxation phenomena that decrease precision. Conductive fibers and fabrics can be made of many kinds of materials, such as polypyrrole or carbon materials (graphene, carbon nanotubes (CNT), etc.). Conductive fibers and fabrics have high sensitivity, but require a complex manufacturing process, and the conductivity of polypyrrole decreases rapidly with time [17]. PVDF film has flexibility, and has excellent sensitivity and dynamic response; however, it exhibits charge leakage phenomena [21]. Regarding nanocomposites, metal-coated CNT-epoxy nanocomposites show high sensitivity in the detection of human respiration and pulse, but it is not suitable to monitor large displacements such as of wrist motion because of the very low stretchability [22]. Generally, the above materials are not ideally suited for the measurement of wrist motion.

Another widely studied class of soft sensor is the dielectric elastomer sensor (DES) that is lightweight, very soft, and has high linearity and stretchability [24]. These characteristics of such a soft sensor make them well suited to measure the motion of human beings as wearable devices. The group of Prof. Anderson has made significant progress with the DES as applied to detect finger motion [25,26,27]. Unlike finger motion, the wrist has three degrees of freedom (DOFs) that are coupled. Due to this coupling, measuring wrist movement is complicated, and as a result previous studies have not applied DESs to the measurement of wrist motion. 

Generally, wearable DESs have an advantage in the measurement of large deformation compared to the above conductive materials, and DESs are more precise and comfortable compared to wearable inertial motion capture sensors. Therefore, in this paper, we demonstrate the use of five wearable DESs to measure wrist motion by utilizing a decoupling method to calculate the component motions, and precision of the DESs can meet the requirements of general motion capture systems.

## 2. Motion Decomposition of Wrist Joint 

**Figure 1:**
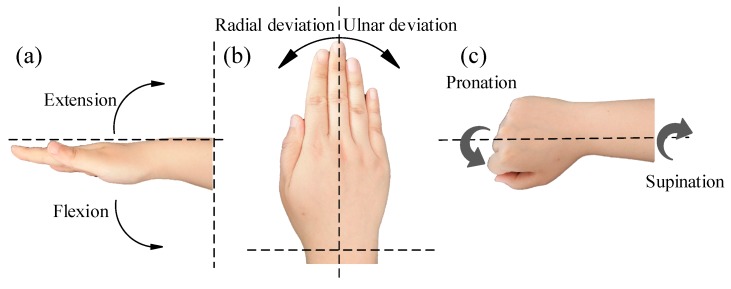
(**a**) Flexion/Extension (F/E); (**b**) Radial/Ulnar deviations (R/U); (**c**) Pronation/Supination (P/S).

The wrist is one of the most important joints in the human body. The complex multi-DOF movements of the wrist joint originate from the ligament, muscular, and skeletal systems. As shown in Figure 1, the wrist can realize six component motions, which are Flexion/Extension (F/E), Radial/Ulnar deviations (R/U), and Pronation/Supination (P/S). The null positions of each component motion are shown in Figure 1. The range of flexion, extension, ulnar deviation, pronation, and supination are 75°, 45°, 40°, 90°, and 90°, respectively. Since the radial deviation motion is very small and rarely used for the majority of people, the angle of the radial deviation motion is less than 10°. Therefore, only the other five component motions are measured in this paper.

## 3. Measurement of the Component Motions of the Wrist Joint 

Based on the analysis given in Section 2, five sensors are needed to measure the five component motions because the DES is quite soft and can only detect stretching. In this section, the fabrication and mounting of the soft sensor are described.

### 3.1. Selection of Materials and Fabrication of the DES

**Figure 2:**
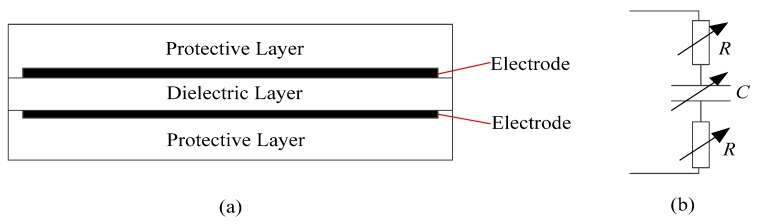
(**a**) Typical structure of the dielectric elastomer sensor (DES); (**b**) Equivalent circuit of the DES.

The typical dielectric elastomer sensor (DES) consists of three layers of DE membranes and two layers of electrodes, as shown in Figure 2a. The middle layer is the dielectric, while the outer layers are used for protection. The protective layers should cover the electrode areas, and also should be as soft as possible so as not to restrict the deformation of the DES. This structure creates an electrically flexible capacitor. The simplified equivalent electrical circuit is a variable capacitor *C* in series with two variable resistors *R*, as shown in Figure 2b.

Both the capacitance and the resistance changes with deformation of the DES, and these changes can be used to infer the deformations. Compared to the series resistance, measuring changes in capacitance with deformation is more straightforward and reliable because the resistance is extremely dependent on the electrode uniformity and environmental temperature [28]. Capacitance of the DES can be calculated by Equation (1):

(1) $$C=\frac{\varepsilon_{0}\varepsilon_{r}A}{d}$$

where $\varepsilon_{0}$ is the vacuum permittivity, $\varepsilon_{r}$ is the relative permittivity of the dielectric layer material, A is the area of the overlapping electrodes, and d is the thickness of the dielectric layer. Variations in strain of the DES can be linearly converted to variations in capacitance, and then linearly converted to an output voltage; therefore, the strain can be calculated by the voltage. A more detailed description of the working principles of the DES can be found in Reference [24].

The dielectric elastomer membrane was fabricated from a two-component silicone solution. The two components were mixed and degassed with the addition of a solvent (isooctane) using a planetary mixer (Thinky ARE-310). The mixture was cast on a substrate (polyethylene terephthalate, PET) using an automatic film coater (ZEHNTNER, ZAA2300) with a thickness determined by a universal applicator (ZEHNTNER, ZUA2000) [29]. The membrane was then allowed to cure in an oven. A graphite-elastomer composite was chosen for the electrode material. The soft electrode consisted of carbon particles (EC300J) dispersed in a soft silicone matrix (MED-4901, Nusil) with a 1:10 mass ratio [30]. MED-4901 has minimum influence on the mechanical performance of the dielectric layer and is difficult to separate from the DES. The electrode was then applied to the dielectric layer using a precise mold and subsequently cured in an oven. A short exposure to oxygen plasma was used to bond the electrode and protective layers together [31]. Finally, lead wires were placed to complete the sensors. Using this method, there were no obvious cracks or discontinuities in the electrode areas. Medical-grade silicone rubber (Bluestar LSR-4305) was chosen as the protective and dielectric material because LSR-4305 has a high tensile strength. The protective layer completely covers all the electrode areas, including fringe field; i.e., the two protective layers were connected at the fringe field. 

### 3.2. Placement of the DES

**Figure 3:**
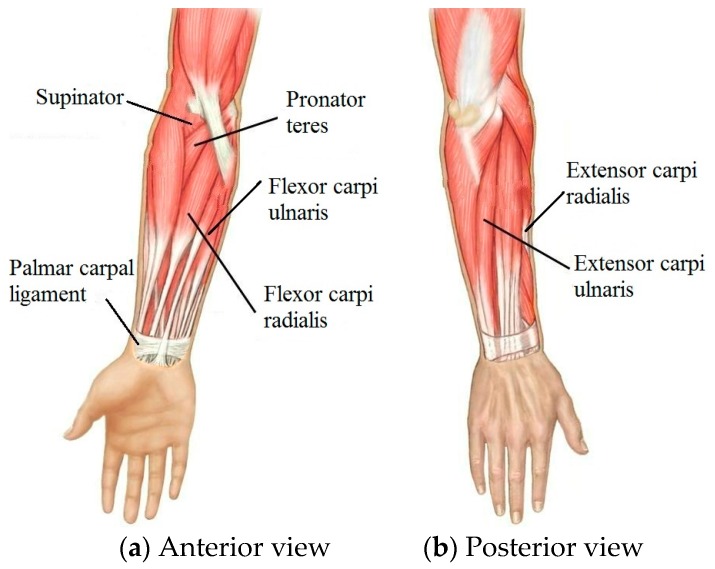
The anatomy of wrist-related muscles: (**a**) Anterior view; (**b**) Posterior view.

The DES should cover the areas where the skin has maximum deformation during wrist motion. These placement areas were determined using the distribution of muscles and tendons because the skin is pulled by these underlying muscles and tendons. Figure 3 shows the anatomy of muscles related to wrist motions. The *flexor carpi ulnaris* (FCU) and *flexor carpi radialis* (FCR) is a muscle group of the human forearm that acts to flex the wrist. The FCU arises from two heads—humeral and ulnar. The FCR runs laterally to the *flexor digitorum superficialis*, inserts into the anterior aspect of the base of the second metacarpal, and has small connections to both the third metacarpal and trapezial tuberosities. *Extensor carpi radialis* (ECR) and *extensor carpi ulnaris* (ECU) is a muscle group of the human forearm that acts to abduct the wrist. The ECR originates from the lateral supracondylar ridge of the humerus and inserts into the dorsal surface of the base of the second metacarpal bone. The ECU originates from the lateral epicondyle of the humerus and the posterior border of the ulna, and crosses the forearm to the ulnar (medial) side to insert at the base of the fifth metacarpal. Ulnar deviation—also known as ulnar drift—relates to the ECU and the FCU, and is also restricted by the palmar carpal ligament. The *pronator teres* works to achieve pronation by pulling on the radius bone of the forearm, which closes to 15° with the arm’s longitudinal axis. The supinator muscle supinates the forearm by pulling on the radius. The supinator consists of two planes of fibers, tipping it −15° from the axis. 

**Figure 4:**
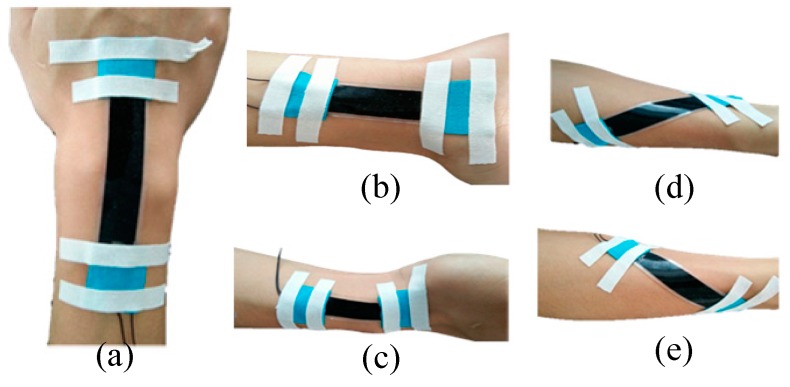
Single motion sensor placement: (**a**) Flexion; (**b**) Extension; (**c**) Ulnar deviation; (**d**) Pronation; (**e**) Supination.

Sliding of the sensors during skin deformation can lead to underestimating the actual motion. To avoid this problem, the DESs were attached to the wrist skin with the aid of Kinesio tape, as shown in Figure 4. Sensors were placed by the anatomical positioning method, and the conformity of the DES to the skin was then assessed. For flexion, the DES was positioned at the three fingers ahead of the styloid process of the ulna and on the middle of the junction of the wrist and palm. The end of the extension DES was placed at the transverse carpal crease, and was situated at the center of the wrist. The ulnar DES begins at the styloid process of the radius and is parallel to the axis of the wrist. The pronation DES originates in the middle of the forearm, and the angle between the pronation DES and the flexion DES is about 15°. In addition, the supination sensor and pronation sensor were symmetrically placed. 

The FCR and FCU flex the wrist, and this muscle group causes the overlying skin to contract in length during concentric muscle contraction. This poses a problem for a DES mounted at this position, as the DES can only measure extensions from its initial unstretched position. Otherwise, the thin DES would buckle. However, the antagonistic ECR and ECU lengthen during the contraction of the FCR-FCU group, so the DESs were placed above the ECR and ECU but parallel to the wrist axis. During the transition from the forearm to the hand, there is a protrusion that affects the close-fitting of the DES to the skin. To avoid this discontinuity, the DES was positioned at the middle of the wrist. The ulnar deviation DES was placed at the palmar carpal ligament. When the wrist pronates, there is a large deformation of the skin above the pronator teres, which is suitable for sensing pronation. Analogously, the DES was placed above the supinator to capture supination. 

### 3.3. Measurement and Analysis of the Component Motion

**Figure 5:**
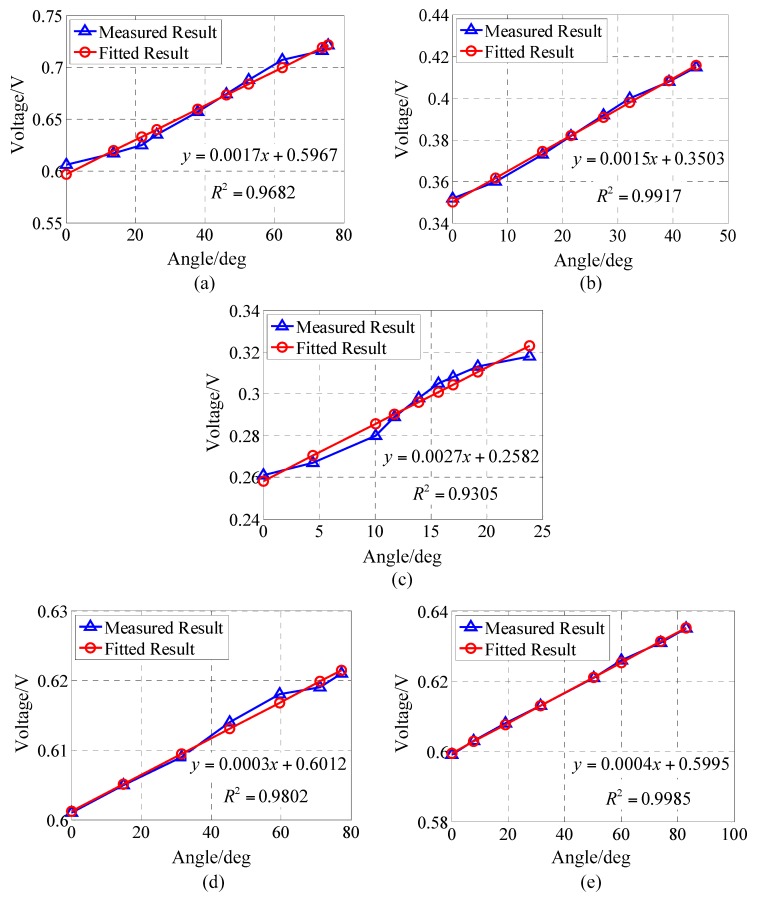
Measurements and linear fitting of wrist motion: (**a**) Flexion; (**b**) Extension; (**c**) Ulnar deviation; (**d**) Pronation; (**e**) Supination.

There were four participants in this study (two men and two women), who were fitted with DESs on their wrists. The maximum wrist size was 7.8 in (19.8 cm) and the minimum wrist size was 5.9 in (15.0 cm). Relationships between the angle of wrist motion and the output voltage of the DES are shown in Figure 5. For all the component motions, the experimental results indicate that the output voltage of the DES was positively related to the joint angle. Linearity errors of flexion, extension, ulnar deviation, pronation, and supination were 6.34%, 3.11%, 7.60%, 5.89%, and 1.82%, respectively.

To evaluate the accuracy of the measurements taken from the DESs, a high-speed camera (Revealer) was utilized, the manufacturer of Revealer is Fuhuang Agile Device and the model name is 2F04. The high speed camera was mounted on a tripod to make the lens perpendicular to the ground. A white substrate (300 mm × 400 mm) was placed and adjusted to face the camera lens, perpendicular to the ground. For all of the component motions, their axis perpendicular to the substrate could be recorded precisely, and the white substrate was used as a background. The camera resolution was 2320 × 1720, and had a high sensitivity (quantum efficiency) of 550 nm. Integrated sensors and field-programmable gate array (FPGA) image acquisition chip completed the image acquisition and preliminary processing tasks. The processing tool (Molysis) of the high-speed camera gets motion parameters by the tracking markers algorithm. The accuracy reached 0.5 pixel, and the pixel size was 7 μm × 7 μm. Based on comparison of the two results, we can calculate the DES measurement error. 

**Figure 6:**
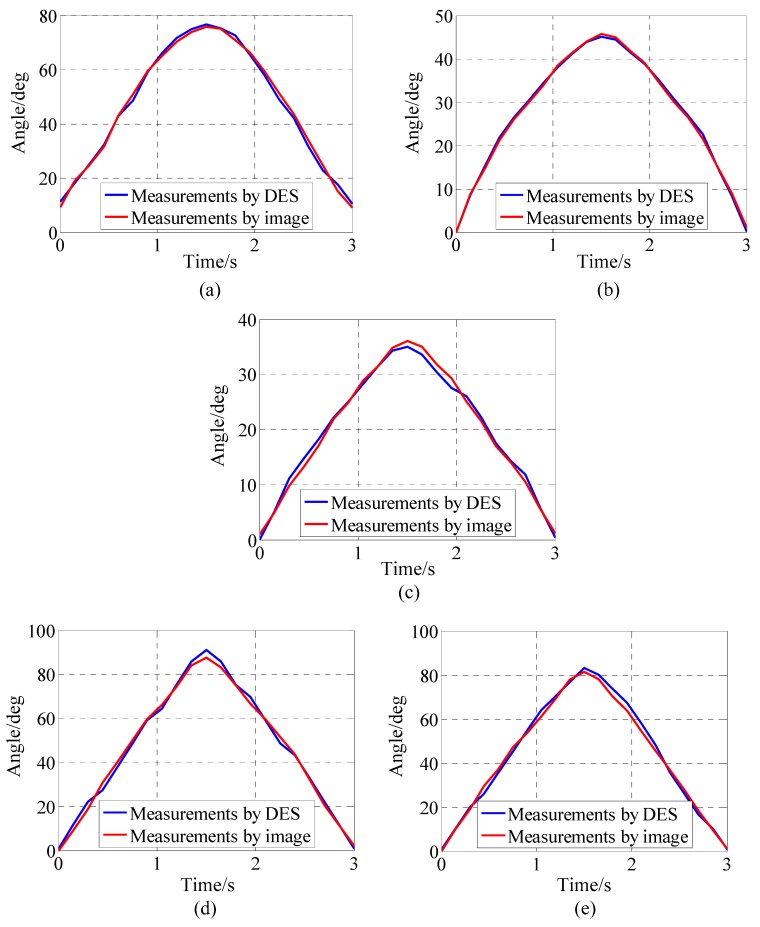
Comparison of measurements by DES and measurements by image processing: (**a**) Flexion; (**b**) Extension; (**c**) Ulnar deviation; (**d**) Pronation; (**e**) Supination.

Figure 6 shows a comparison of the DES measurements with the camera image processing measurements. Due to the high sensitivity of the camera, and because the joint angle image processing error was much smaller than that of the DES, any image processing errors were neglected; i.e., the measurement results of the image processing were regarded as reference values. The maximum errors of flexion, extension, ulnar deviation, pronation, and supination were 2.35°, 1.07°, 2.41°, 3.43°, and 3.44°, respectively. 

## 4. Decoupling of the Measurements of Wrist Motion

### 4.1. Coupling between the Component Motions

**Figure 7:**
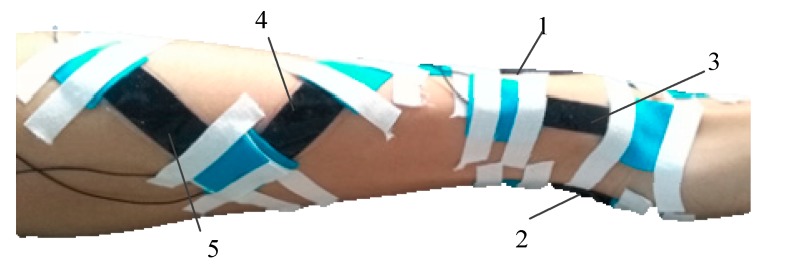
Measurements of the coupling between the component motions.

As mentioned above, the wrist is a joint with multiple DOFs that are coupled with one another. As a result, the component motions of the wrist will act together, affecting the measurements of the DESs. To make the desired measurements, these DOFs should be decoupled. The coupling relationship could be found by moving any component motion from zero position to its extreme position. As shown in Figure 7, five DESs are worn on the wrist and forearm. The wrist moved from its zero position to its extreme position of flexion, extension, ulnar deviation, pronation, and supination, respectively. Average output voltages of three measurements of each component motion are shown in Table 1, and three repetitions are across all subjects in Table 1.

The values along the diagonal in Table 1 are the response of the main sensor (each component motion has a main sensor to detect its variation), and the other values are the coupled output voltages. The most pronounced coupling is the output of DES 3 (the main sensor of ulnar deviation) by flexion. The FCU and FCR act to flex the wrist; meanwhile, the FCU pulls on the ulna bone of the forearm and causes ulnar deviation. So, the ulnar deviation has a strong coupling to flexion. When ulnar deviation occurs, one side of the wrist skin expands and the other side contracts; since the sensor to measure flexion is pulled only on one side, there is no coupling between flexion and extension. Similarly, there is no coupling between flexion and extension or between pronation and supination.

### 4.2. Decoupling Algorithm

As shown in Table 1, the output of the DES is not only affected by the main component motion, but it is also affected by other component motions, and the output voltage can be expressed as

(2) $$y_{i}=k_{i1}\theta_{1}+k_{i2}\theta_{2}+k_{i3}\theta_{3}+k_{i4}\theta_{4}+k_{i5}\theta_{5}+b_{i}\begin{array}{cc} & i=1,2\ldots 5 \end{array}$$

where *i* is the number of DES; 1, 2, 3, 4, and 5 represent flexion, extension, ulnar deviation, pronation, and supination, respectively; $y_{i}$ is the output voltage of DES *i*; and $\theta_{1}$, $\theta_{2}$, $\theta_{3}$, $\theta_{4}$, and $\theta_{5}$ are the rotational angles of each component motion. $k_{i1}$, $k_{i2}$, $k_{i3}$, $k_{i4}$, and $k_{i5}$ are the coupling coefficients of each component motion to DES *i*, and $k_{ij}$ can be obtained by the relationship between the component motion *j* and DES *i*. *b_i_* is a constant that is the output voltage of DES *i* at the zero position of the wrist joint. Experimental results indicate that all the couplings of component motions to the DESs are an approximately linear relationship, so Equation (2) can be written as the following constant coefficient matrix:

(3) $$\left(\begin{array}{l} y_{1}\\ y_{2}\\ y_{3}\\ y_{4}\\ y_{5} \end{array}\right)=\left(\begin{array}{cccc} k_{11} & k_{12} & k_{13} & \begin{array}{cc} k_{14} & k_{15} \end{array}\\ k_{21} & k_{22} & k_{23} & \begin{array}{cc} k_{24} & k_{25} \end{array}\\ k_{31} & k_{32} & k_{33} & \begin{array}{cc} k_{34} & k_{35} \end{array}\\ \begin{array}{l} k_{41}\\ k_{51} \end{array} & \begin{array}{l} k_{42}\\ k_{52} \end{array} & \begin{array}{l} k_{42}\\ k_{53} \end{array} & \begin{array}{l} \begin{array}{cc} k_{44} & k_{45} \end{array}\\ \begin{array}{cc} k_{54} & k_{55} \end{array} \end{array} \end{array}\right)\left(\begin{array}{l} \theta_{1}\\ \theta_{2}\\ \theta_{3}\\ \theta_{4}\\ \theta_{5} \end{array}\right)+\left(\begin{array}{l} b_{1}\\ b_{2}\\ b_{3}\\ b_{4}\\ b_{5} \end{array}\right)$$

Furthermore, Equation (3) can be abbreviated as

(4) Y=K•θ+B

Then, the wrist angle can be calculated from the output voltages of the DESs by

(5) $$\theta =K^{-1}•(Y-B)$$

### 4.3. Example of the Decoupling Algorithm

**Figure 8:**
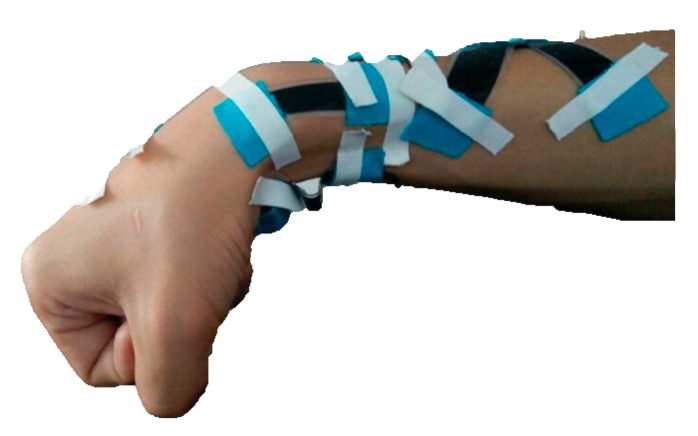
Wrist joint move to a certain position.

Let the wrist joint rotate to the position shown in Figure 8. Then, the output voltages of the DESs were

(6) $$y={\left(\begin{array}{cccc} 0.0052 & 0 & 0.008 & \begin{array}{cc} 0 & 0.018 \end{array} \end{array}\right)}^{T}$$

By experimental measurements, the K coefficient matrix could be obtained:

(7) $$K=\left(\begin{array}{ccccc} 0.0017 & 0 & 0.0003 & 0.0001 & 0\\ 0 & 0.0015 & 0 & 0 & 0.0001\\ 0.0003 & 3 & 0.0027 & 0.0001 & 0.0001\\ 0 & 0 & 0 & 0.0004 & 0\\ 0 & 0 & 0 & 0 & 0.0003 \end{array}\right)$$

By substituting *K* to Equation (5), the angles of the component motions could then be calculated:

(8) $$\theta \;=\;{\left(\begin{array}{cccc} 32.0619 & -3.6579 & -3.1679 & \begin{array}{cc} -4.3616 & 85.2116 \end{array} \end{array}\right)}^{T}$$

Moreover, the joint angles were obtained by image processing as follows:

(9) $$\theta \;=\;{\left(\begin{array}{cccc} 37 & 0 & 0 & \begin{array}{cc} 0 & 90 \end{array} \end{array}\right)}^{T}$$

Compared with the results from the camera’s image processing, the measurement error by the DESs of flexion, extension, ulnar deviation, pronation, and supination were 4.9381°, 3.6579°, 3.1679°, 4.3616°, and 4.7884°, respectively. The error is mainly due to three aspects. First, the relationship between the component motion and the output voltage of the main DES is not precisely linear, and that will introduce an error of about 2.5° when the angle is calculated based on an approximate linear relationship. Second, the coupling of the component motion and the DES is not a linear relationship either, and that will introduce a small error. Finally, the image processing could make an error of several degrees. Generally speaking, the maximum error is not more than 5°, which is acceptable to the majority of requirements of motion capture, so the decoupling algorithm presented here can be applied to measure wrist motion. 

## 5. Discussion

As a strain sensor, experimental results showed that the DES had a high linearity of 0.14%, a high repeatability of 0.81%, a high sensitivity of 0.0082 V/mm, and a low hysteresis of 0.6%; the dynamic response time was about 200 ms. The DES could stretch up to 100% strain. The above characteristics of the DES can meet the potential requirements of wrist motion measurements. 

In measuring wrist motion, a resolution of 1 degree is sufficient in most applications. Moreover, the DES processing circuit can reach a resolution of 0.1 mV, and therefore, the sensitivity of the DES should be greater than 0.1 mV/°. Based on experimental results, the average sensitivities of flexion, extension, ulnar deviation, pronation, and supination were 0.9 mV/°, 0.8 mV/°, 0.5 mV/°, 0.2 mV/°, and 0.2 mV/°, respectively, which is more than enough sensitivity to accurately measure wrist motion in the majority of potential applications. In addition, the results of a pressure test demonstrate that the sensor would be unaffected if the load was less than 50 g. However, when the load is 1000 g, the increment of output voltage is 1.4%. 

The placement of the DESs is a little complex, and their positioning repeatability precision is about 0.5 cm, based on experimental results. This positioning precision is presently lower than the wearable inertial sensor, but the positioning precision can be improved in future designs. This said, considering the variable characteristics of skin and the diversity of the human body, this positioning error is acceptable, and could potentially be reduced if the DES were incorporated into tight-fitting garments.

The decoupling algorithm presented in Section 4.2 can be commonly used, and is not user-specific; however, if the DESs system is worn by different people, the coefficient matrix would change and the system would need to be re-calibrated. The calibration of the coefficient matrix requires sensor positioning, but this requirement is not strict, and half of one centimeter is entirely acceptable. By utilizing the proposed placement method of the DES, a positioning precision of 0.5 cm can be realized. In addition, calibration of the coefficient matrix only need occur one time for each component motion. Based on our experimental results, the measurement repeatability errors of the coefficients *k_ij_* in Equation (3) (the repeatability error *e* of any coefficient *k_ij_* can be calculated by the equation $e=\sqrt{\frac{1}{N-1}\sum_{m=1}^{N}{\left(X_{m}-\mu \right)}^{2}}/\mu$, where N is the number of measurements, *X_m_* is the value of measurement, μ is the average value of all measurements of coefficients *k_ij_*) are less than 7%. This approach to decoupling a complex motion can be utilized when taking measurements of other multi-DOF joints as well, but the coupling matrix should be re-calibrated according to the joint characteristics. However, limited by experimental conditions, 2D measurement was performed in order to validate the decoupling algorithm. A 3D motion evaluation system should be built and applied in the future.

DESs were attached to the wrist skin with the aid of Kinesio tape. If the sensors were integrated into tight-fitting clothing, the relative position of the sensor and the skin would be more precise. The final DES system had the following dimensions: the lengths of flexion, extension, ulnar deviation, pronation, and supination sensors were 60 mm, 35 mm, 25 mm, 60 mm, and 60 mm, respectively. With the exception of the ulnar deviation sensor that had a width of 10 mm, the width of all other sensors was 15 mm. Optimization of these dimensions should be based on the area of skin deformation and the desired sensitivity of the DES. The DES should cover the above area as completely as possible, and the DES should be as wide as possible to maximize the sensitivity. 

In general, compared to traditional methods, measurement using DESs is not limited to indoor spaces. The DES has a very low elastic modulus (only about 0.1 MPa), small thickness (about 0.68 mm), and a high tensile strength (about 3.4 MPa). These characteristics of light weight, softness and flexibility make the DES quite comfortable to wear and move naturally with the human body.

## 6. Conclusions

The wrist has a complex motion and plays a very important role in the action of the hand. Designing a comfortable, portable, and accurate wrist motion capture system can therefore be challenging. In this report, a method to measure wrist motion is proposed based on a DES system. Based on analyzing muscle distribution and skin deformation, five DESs were oriented and affixed to the skin to achieve optimal stretch and sensitivity. In addition, in order to solve the coupling problem of the measurements, a decoupling algorithm was proposed. Based on the decoupling algorithm, complex wrist motion was measured, and the error of all the component motions was less than 5 degrees, well able to meet the requirements of popular motion capture systems.

The dielectric layer and protective layers of the DES were made of medical-grade silicone rubber (Bluestar LSR-4305) that is soft, flexible, and customizable, so the DES can be easily fitted to the surface of the skin and is well-suited to be worn on the human body. This DES system can be applied to wrist rehabilitation training, analysis of athletic performance for training, and virtual reality systems. It can also be used for the control manipulators of robotic systems, or integrated into exoskeleton robotic systems to detect joint motions. Future research will focus on improving the measurement precision and making the system more comfortable to wear.

## Figures and Tables

**Table 1:** Kinematically-coupled output voltages of the five DESs in Figure 7.

The Component Motions	No. 1 Output Variation/V	No. 2 Output Variation/V	No. 3 Output Variation/V	No. 4 Output Variation/V	No. 5 Output Variation/V
Flexion	0.063	0	0.016	0.005	0.002
Extension	0	0.037	0	0.002	0.003
Ulnar Deviation	0.008	0	0.019	0.003	0.002
Pronation	0.004	0.002	0.001	0.016	0
Supination	0.003	0.001	0.002	0	0.017
